# Epilepsy and psychiatric pathologies: A study of a case series

**DOI:** 10.1192/j.eurpsy.2024.989

**Published:** 2024-08-27

**Authors:** E. Smaoui, M. Bouhamed, D. Mnif, F. Cherif, I. Feki, J. Masmoudi

**Affiliations:** Psychiatry department, CHU Hédi Chaker, Sfax, Tunisia

## Abstract

**Introduction:**

Psychiatric pathologies are more common in people with epilepsy than in the general population and have a negative impact on the quality of life of these patients.

**Objectives:**

The objective of this work is to illustrate, through a series of cases, the complex relationship between epilepsy and psychiatric pathologies.

**Methods:**

We report the cases of four patients with different psychiatric pathologies associated with epilepsy admitted to the psychiatry department of Hedi Chaker Sfax. We collected the clinical characteristics of these patients based on their medical files.

**Results:**

The patients were aged 64, 45, 38 and 26 respectively. The first patient had a late-onset vascular epilepsy following the psychiatric pathology onset by 20 years. In the remaining cases epilepsy onset preceded the psychiatric pathology by 6, 3 and 1 year respectively. The aetiology of epilepsy was juvenile myoclonic epilepsy, and idiopathic in 2 cases. The psychiatric pathologies were schizophrenia, obsessive compulsive disorder with schizoid personality, schizoaffective disorder in the bipolar type and mild intellectual disability with histrionic personality, respectively. Familial history of psychiatric disorders was found 2 patients and of epilepsy in one.

**Conclusions:**

Epilepsy and psychosis have a complex and bidirectional relation. Not only are patients with epilepsy at a greater risk of developing a psychotic disorder, but patients with a primary psychotic disorder are also at greater risk of developing epilepsy. The fact that the association between these pathologies is more frequent than expected should prompt more in-depth studies concerning the underlying etiopathogenic mechanisms to improve their management.

**Disclosure of Interest:**

None Declared

